# Effect of soy protein isolate on the functional, pasting, and sensory acceptability of cassava starch‐based custard

**DOI:** 10.1002/fsn3.507

**Published:** 2017-08-19

**Authors:** Toyin E. Akinwale, Taofik A. Shittu, Abdul‐razaq A. Adebowale, Sheriff Adewuyi, Adebayo B. Abass

**Affiliations:** ^1^ Department of Food Technology Federal Institute of Industrial Research, Oshodi, Lagos Lagos Nigeria; ^2^ Department of Food Science and Technology Federal University of Agriculture Abeokuta Ogun State Nigeria; ^3^ Department of Chemical Sciences Federal University of Agriculture Abeokuta Ogun State Nigeria; ^4^ International Institutes of Tropical Agriculture Eastern African Hub Dar es Salaam Tanzania

**Keywords:** cassava starch, custard powder, functional, pasting, soy protein isolate

## Abstract

Fortification of custard powder (CP) with protein from cheap sources such as soybean could potentially improve its nutritive value but may alter its functional and sensory properties. This study was therefore conducted to determine the effect of soy protein isolate (SPI) inclusion (0%–20%) on some functional and sensory properties of cassava starch‐based CP. Functional, pasting, and sensory acceptability were determined using standard methods. Increase in soy protein isolate significantly (*p* < .05) decreased dispersibility, packed bulk density, swelling power, peak, trough, breakdown, final, and setback viscosities, but increased least gelation concentration, water absorption capacity, and solubility index. This study further showed that despite increasing addition of SPI up to 20%, sensory acceptability of the cassava starch‐based CP formulations did not differ significantly, and most of them had very similar acceptability when compared to that of corn starch‐based CP.

## 1 INTRODUCTION

Custard powder is a fine‐textured food product mainly processed from corn starch powder with the addition of flavoring and coloring agents, whereas corn starch is mainly imported to Nigeria with importation cost of about 15 million USD in 2010.

Cassava (*Manihot esculenta* Crantz) is a woody shrub that contains edible root with life cycle of more than 2 years. It is a rich source of energy and it serves as food security and income‐generating crop for millions of people in the developing countries (Amani, Kamenan, Rolland‐Sabaté, & Colonna, 2005), but it is nutritionally deficient because of its low protein content (Montagnac, Davis, & Tanumihardjo, 2009). Cassava starch is produced from cassava root and this starch is applicable in both food and nonfood industries (Singh, Singh, Sharma, & Saxena, 2003).

Soybeans (*Glycine max*) are inexpensive source of protein; they are used as protein supplement in different forms in food combinations. Soy protein isolate is a purified form of soy protein with the exclusion of nonprotein components. They are used to increase protein content and enhance moisture retention (Londhe, Joshi, Bhosale, & Kale, 2011) and they form gels with a good water holding capacity upon heating (Gonçalo, 2012). Soy protein isolate is a complete vegetable protein that contains all the essential amino acids necessary for good health (Burrington, 2000; Liu, 2004). It is used in compounded foods due to its functional properties such as water absorption and for increasing total protein content (Londhe et al., 2011).

Functional and pasting properties are properties that could be used to predict the application and end use of food materials for various food products. The use of cassava starch in various products and manufacturing processes is determined by the functional properties it possess such as gelatinization, pasting, retrogradation, viscosity, swelling, and solubility which varies considerably (Peroni, Rocha, & Franco, 2006; Yuan et al., 2009). The functional properties also depend on the source of starch, presence of various ingredients, and processing conditions. The functional properties of protein are affected by both intrinsic and extrinsic factors. Some of the intrinsic factors are the ratio between hydrophobicity/hydrophilicity as well as the protein's capacity to interact with other components in the food system (Horax, Hettiarachchy, Chen, & Jalaluddin, 2004; Yuan et al., 2009), and some of the extrinsic factors that affect the functionality of proteins are pH, temperature, moisture, and mechanical processing (Kinsella, 1982).

Incorporation of soy protein isolate in custard powder is done to increase its protein content. Okoye (2008) studied the nutrient composition and acceptability of soy‐fortified custard. However, information about the effect of soy protein isolate on the functional, pasting, and sensory acceptability of cassava starch–soy protein isolate custard is scarce in literature. Therefore, the objective of this study was to investigate the effect of soy protein isolate on the functional, pasting, and sensory acceptability of cassava starch‐based custard. This study will provide beneficial information to custard manufacturers and consumers in predicting the behavior of soy protein isolate in custard powder production.

## 2 MATERIALS AND METHODS

### 2.1 Materials

Cassava variety TMS 30572 was harvested at about 15 months from the research farm of the International Institute of Tropical Agriculture (IITA), Mokwa, Niger State, located at the Guinea savannah region of Nigeria. The cassava roots were processed immediately after harvesting into cassava starch powders.

Soy protein isolate was obtained from GNC Foods, Ikeja, Lagos, while vanilla, tartrazine, corn starch custard, and sugar were purchased at Kuto Market, Abeokuta, Ogun State, Nigeria.

### 2.2 Cassava starch extraction

The method described by Oyewole and Obieze (1995) was used for cassava starch extraction.

### 2.3 Formulation of TMS 30572 cassava starch (CS) and soy protein isolate (SPI)

Using simplex lattice design for product formulation which provided eight experimental runs, cassava starch powder and soy protein isolate powder were mixed together and analyzed in the following ratios 100:0, 95:5, 90:10, 85:15, and 80:20 (samples 100:0, 90:10, and 80:20 occurred twice as duplicate from the simplex lattice design used; the analyzed values obtained from these duplicates were almost the same as their counterparts).

### 2.4 Determination of functional properties

#### 2.4.1 Least gelation concentration determination

The method of Coffman and Garcia (1977) was used for least gelation determination.

#### 2.4.2 Packed bulk density determination

The packed bulk density was determined using the method of Waring and Kinsella (1976).

#### 2.4.3 Dispersibility determination

Dispersibility was determined using the method of Kulkarni, Kulkarni, and Ingle (1991).

#### 2.4.4 Water absorption capacity determination

The method described by Lin, Lin, Lin, and Chou (2006) was used for determining the water absorption capacity (WAC).

#### 2.4.5 Swelling power and solubility index determination

The method described by Hirsch and Kokini (2002) was used for swelling power and solubility index determination.

### 2.5 Pasting properties determination

Pasting properties were determined with the Rapid Visco Analyser (RVA) (model RVA 3D+, Network Scientific, Australia) using the method described by Maziya‐dixon, Adebowale, Onabanjo, and Dixon (2005).

### 2.6 Preparation of custard powder

The method of Abdalla, Umsalam, Abdelhalim, and Khalid (2009) with some modifications was used for custard preparation. About 50 g of each sample containing (3.16% vanilla + 0.016% tartrazine) was suspended in 70 ml of water in a plastic container to make thin slurry. Thereafter, 240 ml of boiling water was added to each of the suspended sample with a continuous stirring for 10 min to produce hot gruel. After preparation, 10 g of sugar was added to each of the samples to improve its taste.

### 2.7 Sensory analyses

Untrained 30 members panelists (both male and female) from the Federal University of Agriculture, Abeokuta that were regular consumers of corn starch custard participated in the sensory acceptability test. The participants used were those willing to participate in the acceptability test. The samples of gruel produced were cooled before serving the panelists for determination of the following attributes: appearance, flavor, consistency, taste, and general acceptability on a hedonic scale of 1–9, where 1 = *dislike extremely* and 9 = *like extremely*.

### 2.8 Data analyses

All experimental data obtained were subjected to analysis of variance (ANOVA) procedure of SPSS version 15.0 (SPSS Inc., 2006) at 5% significant level and means were separated using Duncan's multiple range tests.

## 3 RESULTS AND DISCUSSION

### 3.1 Functional properties of TMS 30572 CS powder and SPI blends

**Figure 1:**
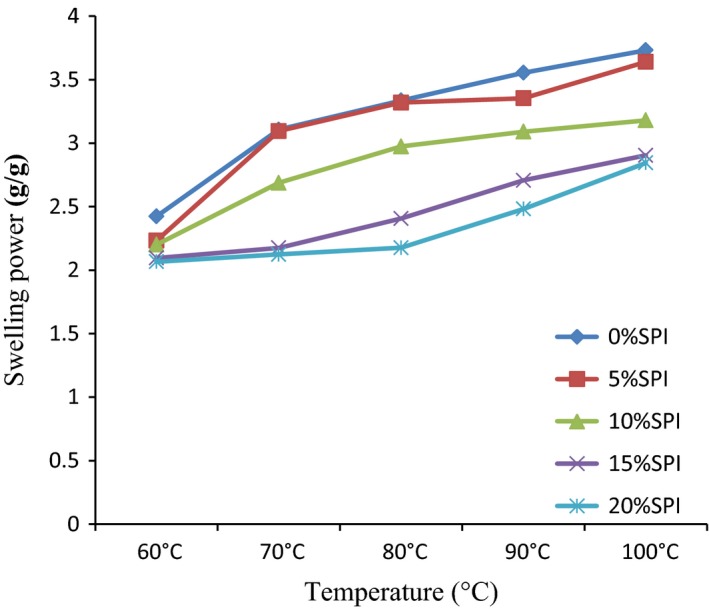
The swelling power of TMS 30572 cassava starch and soy protein isolate blends at different temperature

**Figure 2:**
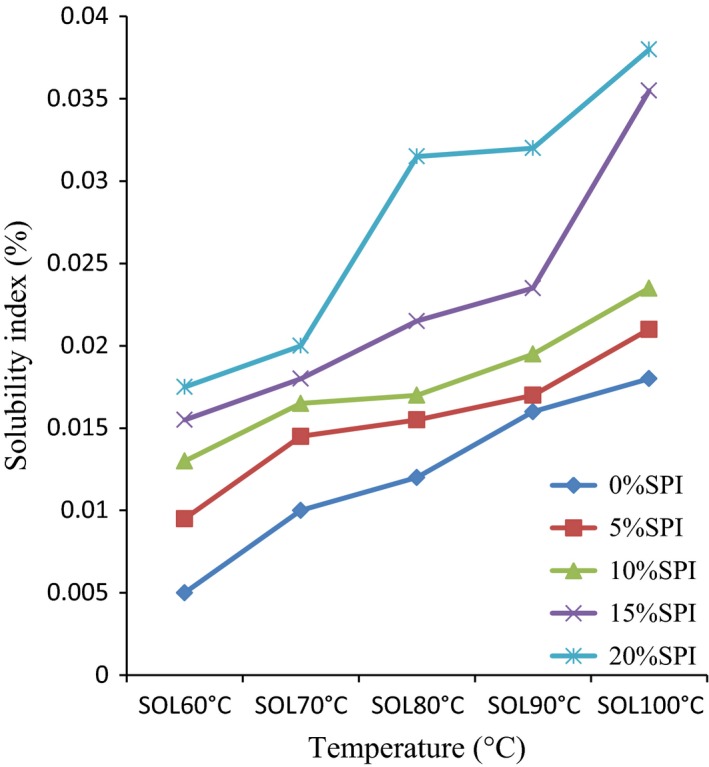
The solubility index of TMS 30572 cassava starch and soy protein isolate blends at different temperature

The functional properties of TMS 30572 and SPI blends are presented in Table 1, Figures 1 and 2. There were significant differences (*p* < .05) in the functional properties of the blends. Functional properties are those parameters that determine the application and end use of food materials for various food products.

**Table 1:** Functional properties, pH, and protein content of TMS 30572 cassava starch (CS) powder and soy protein isolate (SPI) blends

CS:SPI	LGC (%)	Dispersibility (%)	Packed bulk density (g/ml)	WAC (g/g)	pH	Protein (%)
100:0	2.00 ± 0.00^a^	65.5 ± 0.71^d^	0.76 ± 0.02^d^	0.96 ± 0.03^a^	4.69 ± 0.01^a^	0.95 ± 0.13^a^
95:5	5.00 ± 1.41^b^	60.00 ± 0.00^c^	0.42 ± 0.00^c^	0.96 ± 0.06^a^	5.66 ± 0.01^c^	1.53 ± 0.00^b^
90:10	6.00 ± 0.00^bc^	59.00 ± 1.41^c^	0.41 ± 0.01^c^	1.10 ± 0.01^b^	6.13 ± 0.02^d^	6.05 ± 0.13^c^
85:15	7.00 ± 1.41^bc^	58.00^a^ ± 0.00^bc^	0.39 ± 0.01^c^	1.16 ± 0.02^b^	6.37 ± 0.02^e^	8.02 ± 0.13^d^
80:20	8.00 ± 0.00^c^	56.00 ± 0.00^b^	0.37 ± 0.00^b^	1.34 ± 0.05^c^	6.58 ± 0.00^f^	9.99 ± 0.25^e^
100:0 (R)	3.00 ± 1.41^a^	65.00 ± 1.41^d^	0.77 ± 0.00^d^	0.97 ± 0.01^a^	4.72 ± 0.03^b^	1.09 ± 0.00^ab^
90:10 (R)	6.00 ± 0.00^bc^	59.00 ± 1.41^c^	0.41 ± 0.01^c^	1.10 ± 0.01^b^	6.14 ± 0.01^d^	5.84 ± 0.25^c^
80: 20 (R)	8.00 ± 0.00^c^	56.00 ± 0.00^b^	0.37 ± 0.00^b^	1.33 ± 0.03^c^	6.58 ± 0.01^f^	9.77 ± 0.33^e^

Values are mean ± standard deviation. Mean values (*n* = 2) having different superscript alphabets in the same column are significantly different (*p* < .05). CS, cassava starch; SPI, soy protein isolate; LGC, least gelation concentration; WAC, water absorption capacity, (R), duplicate of trial according to design.

The least gelation concentration (LGC) of the blends ranged from 2.00% to 8.00%. LGC is the least quantity of starch blends required to form a gel. There was increase in LGC as SPI inclusion increased with 100% CS having the least mean value, while 80% CS:20% SPI had the highest mean value. The reported LGC values showed that the inclusion of SPI affected the gelling ability of the blends. This may be due to the lower gelling ability of SPI in comparison with CS powder, and this implies that more blends will be required to form a gel. This supports the findings of Ogunwolu, Henshaw, Mock, Santros, and Awonorin (2009) who reported that higher concentration of protein is often required for gelation.

Packed bulk density ranged from 0.37 to 0.77 g/ml. Packed bulk density is a parameter that determines the ease of transportation and packaging of powdery products. Increase in SPI inclusion caused a decrease in the bulk density of the blends with 100% CS having the highest mean value, while 80% CS:20% SPI had the least mean. This probably suggests that 80% CS:20% SPI blend will occupy less space during packaging and more products can be transported.

Dispersibility ranged from 56.00% to 65.50%. Dispersibility is a property that indicates the rate of reconstitution of flour sample in water. Increase in SPI caused decrease in dispersibility of the blends with 100% CS having the highest mean value, while 80% CS:20% SPI had the least mean value. It was observed that the lower the dispersibility value, the higher the formation of lumps in the gruels, this supports the findings of Akinwale et al.(2016) who reported that lower powder dispersibility value indicates higher tendency to form lumps in gruel. The decrease in dispersibility of fortified CS‐based custard as SPI inclusion increased may be due to the disparity in the rate of water absorption by SPI and CS powder. Also, SPI swells and increases its volume at room temperature. This imbalance in the rate of absorbing water and the swelling occurrence of SPI at room temperature may be the cause of this lump formation, but if immediate energy is applied at the beginning of the mixing period to enhance homogeneity, this inconsistency causing lump formation may be prevented or reduced.

Water absorption capacity measures the amount of water held by the protein matrix at room temperature. Water absorption capacity ranged from 0.96 to 1.34 g/g, 100% CS had the lowest and 80% CS:20% SPI had the highest mean value. The observed increase in WAC is probably due to the increase in SPI inclusion because of its hydrophilic sites which has good affinity for absorbing water. This supports the findings of Otegbayo, Samuel, and Alalade (2013) who reported that as substitution of soy in tapioca increases, there is increase in the hydrophilic sites causing more water to be absorbed.

The swelling power and solubility index of TMS 30572 CS and SPI blends at different temperatures are presented in Figures 1 and 2, respectively.

The swelling power increased with increase in temperature and it slightly decreased with increase in SPI inclusion. The increased swelling power of the blends with increase in temperature is in agreement with the findings of Agunbiade and Longe (1999) and Adebowale, Sanni, and Fadahunsi (2011). It is likely that the force of attraction between the starch molecules was weakened by the increase in temperature, thereby enhancing more water uptake by the starch granules and allowing more swelling effect. Also, the slight decrease in swelling power as SPI addition increased in this study may be due to the dilution effect of SPI which had lower gelling ability in comparison with CS powder and also the interaction between the starch and protein as a result of the opposite charges and this restricts the swelling. This is in agreement with the findings of Shimelis, Meaza, and Rakshit (2006).

Solubility index increased with increase in temperature, but decreased with increase in SPI inclusion. Solubility is the percentage of sample dissolved in a solvent. The increase in solubility index with increase in temperature observed in fortified CS‐based custard support the findings of Bolontrade, Scilingo, and Añón (2013) who worked on legume proteins. This may be due to the dissociation of the quaternary structure of SPI, thereby releasing smaller peptides and increasing the solubilization of the protein (Rangavajhyala, Ghorpade, & Hanna, 1997).

Also, increase in solubility index of the fortified custard paste as SPI inclusion increased observed in this study may be due to the SPI's pH which is above the isoelectric point of protein. Previous work has expressed increase in solubility of material when solution is maintained above the isoelectric point (Adebowale, Sanni, & Awonorin, 2005; Kanu et al., 2007a,b; Lee, 2011; Pelegrine & Gasparetto, 2005; Philip et al., 2007). This is probably due to the occurrence of excess charges of the same sign which produce electrostatic repulsive forces among the protein molecules (Adebowale et al., 2005; Pelegrine & Gasparetto, 2005). This electrostatic repulsive force between the positive charges of the fortified CS‐based custard probably keeps the charges apart, allowing the protein configuration to be more open and allowing more water to interact with the protein molecules.

### 3.2 Pasting properties of blends of TMS 30572 CS powder and SPI

Pasting properties of blends of TMS 30572 CS and SPI is presented in Table 2. There was significant difference in the pasting profile except peak time and pasting temperature (*p* < .05). Increase in SPI inclusion caused reduction in the peak viscosity, trough viscosity, breakdown viscosity, final viscosity, and setback, the values ranged from 155.38 to 279.42 RVU, 75.21 to 117.63 RVU, 78.25 to 161.79 RVU, 98.67 to 153.88 RVU, and 24.46 to 36.25 RVU, respectively.

**Table 2:** Pasting properties of TMS 30572 cassava starch powder and soy protein isolate (SPI) blends

CS:SPI	Peak viscosity (RVU)	Trough viscosity (RVU)	Breakdown viscosity (RVU)	Final viscosity (RVU)	Setback viscosity (RVU)	Peak time (min)	Pasting temperature (°C)
100:0	279.42 ± 10.02^f^	117.63 ± 3.47^e^	161.79 ± 13.49^d^	153.88 ± 5.37^f^	36.25 ± 1.88^d^	4.04 ± 0.23^a^	75.93 ± 1.17^b^
95:5	236.96 ± 3.83^e^	112.96 ± 1.59^e^	124.00 ± 5.42^c^	145.38 ± 1.83^f^	32.42 ± 0.23^c^	4.20 ± 0.00^a^	75.88 ± 0.11^b^
90:10	200.92 ± 1.18^d^	99.04 ± 5.36^d^	101.88 ± 4.18^b^	128.67 ± 4.24^e^	29.63 ± 1.12^bc^	4.24 ± 0.05^a^	77.10 ± 0.57^b^
85:15	172.54 ± 3.59^c^	85.46 ± 7.48^bc^	87.08 ± 3.89^a^	112.84 ± 8.01^cd^	27.38 ± 0.53^ab^	4.20 ± 0.00^a^	76.78 ± 0.04^b^
80:20	155.38 ± 5.13^b^	77.13 ± 4.66^b^	78.25 ± 0.47^a^	101.88 ± 4.89^bc^	24.75 ± 0.24^a^	4.20 ± 0.09^a^	76.30 ± 1.69^b^
100:0 (R)	265.83 ± 9.19^f^	116.92 ± 4.48^e^	148.92 ± 4.72^d^	152.71 ± 7.01^f^	35.79 ± 2.53^d^	4.17 ± 0.49^a^	76.78 ± 0.04^b^
90:10 (R)	193.08 ± 9.89^d^	89.75 ± 7.77^cd^	103.33 ± 2.12^b^	120.46 ± 7.37^de^	30.71 ± 0.41^c^	4.20 ± 0.09^a^	76.68 ± 0.04^b^
80: 20 (R)	155.67 ± 2.71^b^	75.21 ± 0.88^b^	80.96 ± 2.89^a^	98.67 ± 1.06^b^	24.46 ± 1.59^a^	4.17 ± 0.05^a^	75.68 ± 0.25^b^

Values are mean ± standard deviation. Mean values (*n* = 2) having different superscript alphabets within the same column are significantly different (*p* < .05). CS, cassava starch; SPI, soy protein isolate; (R), duplicate of trial according to design.

Peak viscosity is the highest viscosity attained during starch gelatinization. It is an indication of the extent of starch swelling before its physical breakdown. This probably suggests that as SPI inclusion of the fortified CS‐based custard increased, it interfered with the swelling ability of the fortified CS‐based custard probably because SPI has good water affinity and will compete with the water the starch needs for swelling to occur, thus hindering the extent of swelling.

Final viscosity is an indication of the paste stability after cooking and cooling. It indicates the viscous nature of the paste after cooling. The decrease observed in the final viscosity of the fortified CS‐based custard gruel as SPI inclusion increased, probably suggests that 80% CS:20% SPI inclusion may be desirable for infant consumption because of the less dense paste viscosity.

Breakdown viscosity is used to determine the stability of starch paste and high breakdown value indicates reduced starch paste stability. Increase in SPI inclusion led to decrease in the breakdown viscosity of the fortified CS‐based custard. This probably suggests that increase in SPI inclusion will increase the stability of the custard gruel.

Setback is also a condition that affects the stability of starch paste. Increase in SPI inclusion led to decrease in setback viscosity of fortified CS‐based custard. This probably suggests that fortified custard will have higher resistance to retrogradation and syneresis effect may be reduced, as reported that high setback viscosity indicates lower resistance to retrogradation (Sanni & Ayinde, 2002). This may be due to the slower realignment of the starch molecules in the blends because of the reduced amylose content, thereby causing reduced retrogradation effect due to its higher resistance to retrogradation.

The similarity observed in the peak time and pasting temperature of the fortified CS‐based powders probably implies that they required almost the same duration and temperature, respectively, for preparation into paste.

Table 3 shows the Pearson's correlation matrix between pasting properties of variety TMS 30572 fortified cassava starch‐based custard, protein, and pH. Inverse relationship was observed between protein and the pasting profile of fortified CS‐based custard (peak viscosity, trough viscosity, final viscosity, breakdown viscosity, and setback). This may be due to the limitation of the starch access to water by the protein, thereby reducing the gelatinization ability of the starch. This is in agreement with the findings of Aprianita, Purwandari, Watson, and Vasiljevic (2009) as well as Moorthy (2002) who reported that high protein content negatively affected the pasting properties of starchy food products.

**Table 3:** Pearson's correlation matrix between pasting properties of variety TMS 30572 fortified cassava starch‐based custard, protein, and pH

	Peak viscosity	Trough viscosity	Breakdown viscosity	Final viscosity	Set back viscosity	Protein	PH
Peak viscosity	1.00						
Trough	0.96^a^	1.00					
Breakdown	0.99^a^	0.91^a^	1.00				
Final	0.97^a^	0.99^a^	0.92^a^	1.00			
Setback	0.95^a^	0.93^a^	0.93^a^	0.95^a^	1.00		
Protein	−0.97^a^	−0.97^a^	−0.94^a^	−0.97^a^	−0.94^a^	1.00	
pH	−0.97^a^	−0.91^a^	−0.98^a^	−0.92^a^	−0.93^a^	0.93^a^	1.00

a Correlation is significant at the 0.01 significant level (two‐tailed).

### 3.3 Sensory properties of blends of TMS 30572 CS powder and SPI

Sensory attributes of blends of TMS 30572 CS and SPI custard in comparison with corn starch custard is presented in Table 4. There was significant difference in the sensory attributes of corn starch custard and custards from cassava–soy protein isolate blends (*p* < .05), except flavor and consistency. Sensory evaluation measures, analyses, and interprets human reactions to characteristic of foods and materials perceived by the senses of sight, smell, taste, touch, and hearing (Stone & Sidel, 1993).

**Table 4:** Sensory properties of blends of TMS 30572 cassava starch powder and soy protein isolate (SPI)

CS:SPI	Appearance	Flavor	Consistency	Taste	Overall acceptability
100:0	6.26 ± 1.66^a^	5.93 ± 1.70^**a**^	5.26 ± 1.38^**a**^	5.66 ± 1.34^a^	6.06 ± 1.90^a^
95:5	5.80 ± 1.08^a^	6.80 ± 1.20^**a**^	6.26 ± 1.22^**a**^	5.86 ± 0.91^a^	6.33 ± 0.89^ab^
90:10	5.93 ± 1.03^a^	6.26 ± 1.16^**a**^	5.66 ± 1.11^**a**^	5.66 ± 1.34^a^	5.93 ± 1.33^a^
85:15	6.26 ± 0.70^a^	6.60 ± 1.05^**a**^	6.00 ± 1.77^**a**^	5.93 ± 1.53^a^	6.86 ± 1.55^ab^
80:20	6.13 ± 0.83^a^	6.46 ± 0.83^**a**^	5.86 ± 0.99^**a**^	5.73 ± 1.03^a^	6.40 ± 0.98^ab^
100:0	5.73 ± 2.05^a^	6.00 ± 1.51^**a**^	5.93 ± 1.27^**a**^	5.46 ± 1.30^a^	5.86 ± 1.35^a^
90:10	6.06 ± 1.43^a^	6.00 ± 1.46^**a**^	6.12 ± 1.20^**a**^	6.06 ± 0.85^a^	6.56 ± 1.26^ab^
80:20	6.41 ± 1.22^a^	6.82 ± 1.46^**a**^	6.00 ± 1.41^**a**^	6.23 ± 1.25^a^	6.52 ± 1.17^ab^
Corn starch custard	7.50 ± 1.00^b^	6.83 ± 1.64^**a**^	6.25 ± 0.75^**a**^	7.33 ± 1.37^b^	7.41 ± 1.24^b^

Values are mean ± standard deviation. Mean values having different superscript alphabets within the same column are significantly different (*p* < .05). CS, cassava starch.

Appearance is an important attribute because of its influence in gruel acceptability. The mean score of the gruel's appearance is presented in Table 4. Significant difference (*p* < .05) was observed in the appearance of the gruels. The samples score was rated between 7.50 and 5.73 on 9‐point hedonic scale with corn starch custard having the highest rating, while 100% CS had the least appearance. The higher rating for corn starch custard may be as a result of the yellowish nature of the corn starch.

Flavor of a food product is also an important determinant in its acceptance. There was no significant difference (*p* > .05) in the flavor of the gruels. All the samples had close similarities in their flavor acceptance.

Consistency is also an important determinant of gruel acceptability. Significant difference (*p* > .05) was not observed in the consistency of the gruels. The mean score for the custard's consistency ranged between 6.25 and 5.26. Corn starch custard had the highest rating, while 100% CS had the least rating.

Taste is another attribute that determines the acceptance of a food product. Significant difference (*p* < .05) was observed in the taste of the gruels. The mean score for the taste perception by the panelists ranged between 7.33 and 5.46 with corn starch custard having the highest mean score, while 100% CS had the least mean score.

The overall acceptability of the custard gruel ranged between 7.41 and 5.86. Corn starch custard which is the control sample had higher rating by the panelists than the cassava–soy protein isolate custard. It could be due to the fact that the panelists are used to corn starch custard as well as the difference in the chemical composition of the raw materials used. This is in support with the findings of Nwokeke, Adedokun, and Osuji (2013).

## 4 CONCLUSIONS

This study has shown that increased SPI inclusion caused decreased dispersibility, peak, breakdown, setback, and final viscosities of the custard powder. The lower setback induced by SPI is an improvement in paste stability. There were also indications from lower final viscosities (less density); this could make cassava starch custard fortified with 20% SPI suitable as weaning food for children.

Although, corn starch custard had higher sensory rating than cassava–soy protein isolate custards, the fortified custard samples were acceptable with up to 20% SPI inclusion.

## CONFLICT OF INTEREST

None declared.
